# Our Journal for 1882

**Published:** 1882-01-20

**Authors:** 


### EDITORIALS AND MISCELLANEOUS.

### *OUR JOURNAL FOR* 1882.

We are pleased to announce to the friends of THE RECORD that while very few have discontinued the Journal the present year, that many additions to our list are coming in, and that the outlook for the future is very encouraging. We trust that our subscribers will work for the Journal. A kind word aptly spoken will often be sufficient to procure us a new subscriber.

We trust also that our friends will write for us. Will be especially thankful for short, practical items, facts, formulæ and reports of cases.

